# Prevalence of knee pain, radiographic osteoarthritis and arthroplasty in retired professional footballers compared with men in the general population: a cross-sectional study

**DOI:** 10.1136/bjsports-2017-097503

**Published:** 2017-11-03

**Authors:** Gwen Sascha Fernandes, Sanjay Mukund Parekh, Jonathan Moses, Colin Fuller, Brigitte Scammell, Mark Edward Batt, Weiya Zhang, Michael Doherty

**Affiliations:** 1 Division of Rheumatology, Orthopaedics and Dermatology, School of Medicine, University of Nottingham, Nottingham City Hospital, Nottingham, UK; 2 Arthritis Research UK Centre for Sports, Exercise and Osteoarthritis, Queens Medical Centre, Nottingham, UK; 3 Arthritis Research UK Pain Centre, Nottingham City Hospital, Nottingham, UK; 4 Nottingham University Hospitals NHS Trust, Queens Medical Centre, Nottingham, UK; 5 Colin Fuller Consultancy Ltd, Sutton Bonnington, UK

**Keywords:** osteoarthritis, football, epidemiology

## Abstract

**Objectives:**

To determine the prevalence of knee pain, radiographic knee osteoarthritis (RKOA), total knee replacement (TKR) and associated risk factors in male ex-professional footballers compared with men in the general population (comparison group).

**Methods:**

1207 male ex-footballers and 4085 men in the general population in the UK were assessed by postal questionnaire. Current knee pain was defined as pain in or around the knees on most days of the previous month. Presence and severity of RKOA were assessed on standardised radiographs using the Nottingham Line Drawing Atlas (NLDA) in a subsample of 470 ex-footballers and 491 men in the comparison group. The adjusted risk ratio (aRR) and adjusted risk difference (aRD) with 95% CI in ex-footballers compared with the general population were calculated using the marginal model in Stata.

**Results:**

Ex-footballers were more likely than the comparison group to have current knee pain (aRR 1.91, 95% CI 1.77 to 2.06), RKOA (aRR 2.21, 95% CI 1.92 to 2.54) and TKR (aRR 3.61, 95% CI 2.90 to 4.50). Ex-footballers were also more likely to present with chondrocalcinosis (aRR 3.41, 95% CI 2.44 to 4.77). Prevalence of knee pain and RKOA were higher in ex-footballers at all ages. However, even after adjustment for significant knee injury and other risk factors, there was more than a doubling of risk of these outcomes in footballers.

**Conclusions:**

The prevalence of all knee osteoarthritis outcomes (knee pain, RKOA and TKR) were two to three times higher in male ex-footballers compared with men in the general population group. Knee injury is the main attributable risk factor. Even after adjustment for recognised risk factors, knee osteoarthritis appear to be an occupational hazard of professional football.

## Introduction

Football is the world’s most popular team sport. Worldwide over 265 million people are estimated to play football and of these 1 10 000 are professional male footballers.^1^ The average career of a professional footballer lasts 13.5 years and, despite typically being extremely fit, engaging in high-intensity match-play and training can result in sport-related health risks.^2^ Professional football has a high injury rate and 17% of all injuries involve the knee.^3^ Apart from overt acute injury, the cumulative effect of repetitive microtrauma and joint overloading could also prove deleterious to the knee joint, as is recognised in other physically demanding occupations such as coal mining.^4^

Knee osteoarthritis (KOA) is a common complex disorder with multiple risk factors including injury.^2^ Although there have been long-standing concerns over the risk of KOA in ex-footballers, only six studies have examined this association.^5–10^ However, these studies used different definitions of KOA and often focused on just one or two outcomes (eg, knee symptoms, structural KOA on imaging, self-reported diagnosis of KOA). Most studies were small, four had no comparison groups and adjustment for other known risk factors for KOA was absent or limited.^2^ Indeed, a recent systematic review cited the same limitations and concluded that only ‘very low quality’ evidence suggests football increases the risk of KOA.^11^ No studies have used a general population group for comparison, so whether ex-footballers have more KOA than the general population remains unknown.

We hypothesise that, when recognised risk factors are adjusted for, several outcomes relevant to KOA, including knee pain, radiographic KOA (RKOA), radiographic chondrocalcinosis (CC) (this co-associates with KOA and has been associated with prior joint trauma^12^), physician-diagnosed KOA and knee arthroplasty (a surrogate for clinically severe KOA) are more prevalent in ex-footballers compared with the general population. Therefore, we undertook the following cross-sectional study comparing male ex-footballers with men in the general population.

The objectives of this study were:

1. To determine the prevalence of KOA outcomes (specifically knee pain, RKOA, CC, requirement for knee replacement surgery (TKR)) and risk factors for knee OA in a sample of retired male professional footballers in the UK and in a random sample of men in the general population.
2. To compare the prevalence of these outcomes in ex-footballers and the general population with adjustment for other known risk factors.
3. To determine the main attributable risks for any increased prevalence in knee OA outcomes in ex-footballers.

## Methods

A cross-sectional design was used, involving postal questionnaire surveys to ex-footballers and to a sample of men in the general population to gain information on knee pain, knee surgery and KOA risk factors as well as simple demographics, occupational history, general health and current medications. Participants who indicated interest in attending for knee radiographs, irrespective of knee pain status and who provided written informed consent, were invited to undergo bilateral knee radiographs to determine RKOA and CC.

### Participants

Ex-footballers were recruited via the Professional Footballers’ Association (PFA) and former players’ associations (n=21 professional clubs). Inclusion criteria for ex-footballers were men aged over 40 years who had played professionally (in the top four tiers of the English Football League). The comparison group were recruited from the Knee Pain and Related Health in the Community Study (KPIC), involving recruitment via 12 general practitioner/family medicine (GP) practices in the UK Midlands region. All men on these UK National Health Service GP registers aged 40 years and older who were not terminally ill, were able to give written informed consent and had no other reason judged by the GPs to exclude them from the study were sent the questionnaire.

### Questionnaire survey

The postal questionnaire was developed based on previously published questionnaires.^13 14^ Through public and patient involvement, two pilot versions were evaluated to identify any problems with content, language and layout.

The 44 775 questionnaires were similarly constructed to capture detailed information about the participant, their medical history and putative risk factors for KOA.^5^ A validated screening question was used to determine presence of current knee pain: "Have you ever had knee pain for most days of the past 1 month?”^14^ Additionally, a body pain mannequin^15^ was used to locate pain in other body regions. There were specific enquiries about comorbidities, current medications and any past knee surgery including TKR. Constitutional knee alignment (in early 20s), current knee alignment and the index-to-ring finger length ratio (2D:4D) were assessed using validated line drawings.^16 17^ These drawings, which illustrated the direction and severity of each alignment grade, allowed participants to choose their knee alignments grades separately for early adult life and for current alignment. The grades being: A=severe varus, B=mild varus, C=straight legs, D=mild valgus and E=severe valgus. The 2D:4D ratio can be visually classified as pattern 1 (index longer than ring finger), pattern 2 (index length equal to ring finger) or pattern 3 (index shorter than ring finger) and it is pattern 3 that has been associated with RKOA.^17^ Nodal OA was determined using a validated diagram^18^ and classified as present in those reporting nodes on at least two rays of both hands.^19^ Significant knee injury was defined as ‘*one which caused pain for most days for at least a 3-month period and resulted in an absence from all training and matches during this time’*. Occupations were classed as ‘high risk for KOA’ based on published evidence.^20^ Each listed occupation per individual was analysed and the data dichotomised into high-risk or low-risk groups (excluding professional football careers).

### Radiographic knee osteoarthritis and chondrocalcinosis

All ex-footballers who returned a questionnaire indicating willingness to have knee radiographs, and who lived within 40 miles of a Spire Healthcare Hospital were invited to attend. Similarly, all general population men in KPIC who had indicated interest were invited to attend the Nottingham University Hospitals Radiology Department for bilateral knee radiographs. Participants who had undergone bilateral TKR were excluded from radiographic assessment. All radiographs (weight-bearing semi-flexed posterior-anterior and 30° flexion skyline views) were performed using published protocols.^16 21^ Radiographs were scored by one observer (GSF), blinded to knee pain status, as a single mixed batch using HIPAX Dicom software. The Nottingham Line Drawing Atlas (NLDA) is a logically derived, interval (not ordinal)-based atlas and was used to score individual compartment and composite joint space narrowing (JSN), individual compartment and subsequent composite osteophyte and combined (global JSN and osteophyte) scores for each knee.^21^ The NLDA uniquely provides separate illustrations for JSN for men and women to account for the normally wider joint space width in men and gives 0–5 interval scores for osteophyte at eight sites in the three compartments with consideration for natural variations in osteophyte shape. Separate line drawings for joint space width and osteophyte removes the distraction of combined features and replaces the ordinal grading inherent in photographic atlases. The Kellgren and Lawrence (KL) composite grade (0–4) was also scored for the combined tibiofemoral compartments (medial and lateral) and separately for combined patellofemoral compartments (medial and lateral) using verbal descriptors for features of OA (JSN and/or osteophyte presence) as opposed to a photographic atlas.

Definite dichotomised RKOA using the NLDA was defined as definite JSN (score >2) and definite osteophyte (score >2) in any compartment. CC in either hyaline or fibrocartilage was defined as present or absent. RKOA using KL was defined as grade >3 (definite osteophyte and definite narrowing) and additionally, grade >2 (definite osteophyte and possible narrowing) in any compartment. Intra-observer agreement (GSF) and interobserver agreement (GSF and AS) were assessed for NLDA and KL grades using kappa coefficients.

### Statistical analyses

Based on a 15% prevalence of RKOA in the general population,^22^ the sample size needed was calculated using the z test and a logistic regression model with an a priori type power analysis. We assumed our data would include multiple confounding factors with a multiple correlation coefficient of 0.3 between them. With a power of 80% at 0.05, one tail (assuming footballers would have greater risk of knee OA than the general population) and a relative risk (RR) of 2.0, the sample size required was 424 participants per group. The power calculations were done using GPower, V.3.1.9.2.

Categorical variables were reported as frequencies and percentages and continuous variables as means and SD. To determine whether distributions of the variables were statistically significantly different between ex-footballers and the general population, a t-test (continuous variables) or Χ^2^ test (categorical variables) was used. Statistical significance was defined as p<0.05.

The RR, that is, the ratio of the prevalence of knee OA outcomes between the ex-footballers versus that in the general population was calculated. A multivariable logistic regression model was used to adjust for other confounding factors such as age, body mass index (BMI) and injury. This was followed by the adjrr Stata command.^23^ RR was determined instead of ORs as the outcomes of interest, such as KP and RKOA are not rare and therefore the use of ORs would inflate the estimate. The primary purpose of this analysis was to confirm whether playing professional football is a risk factor independent from other potential knee OA risk factors. We therefore ran four analyses to follow this up. First, we calculated crude RR for playing professional football without any adjustment. Second, we estimated the RR adjusted for two common confounders for OA, age and BMI (model 1). Third, we brought injury into the model as it is a major risk factor of OA for anyone in the general population to examine whether footballing per se without a major injury is a risk factor for knee OA (model 2). Finally, we included all putative risk factors/confounders previously established in the literature and collected in the study (age, BMI, nodal OA, 2D:4D ratio, alignment, injury, high-risk occupation and comorbidities).^24^ We also calculated the risk difference (RD) as an absolute measure of association according to the STROBE.^25^ While the RR is the ratio of knee OA outcomes between the ex-footballers and controls, the RD is the difference between the prevalence of knee OA outcomes between these groups. The RD was calculated using a logistic regression model for each knee OA outcome (dichotomous) between the ex-footballers and general population followed by the same adjrr Stata command. The RR and RD in cross-sectional studies have also been termed as prevalence proportion ratio (PPR) and prevalence proportion difference (PPD), respectively to differ from those measured in cohort studies where the incidence rather than prevalence is used.^26^ However, despite the different terminologies, the calculations are identical, that is, RR or PPR=r1/r2, whereas RD or PPD=r1–r2, where r1 is the risk (prevalence in cross-sectional study or incidence in cohort study) in the exposure group (eg, ex-footballers) and r2 is the risk in the non-exposure group (eg, general population control). We therefore opted to use the RR and RD for this paper as they are more commonly used terms for relative risk and attributable risk and understood by the majority of clinicians. We had very few missing data at random (eg, where BMI was not reported by a participant). Imputation or modelling was therefore not undertaken for the occasional missing values.

All analysis was conducted using Stata IC V.14 on Windows 7 Operating System and power calculations undertaken using Power and Precision V.2.1.

## Results

**Figure 1:**
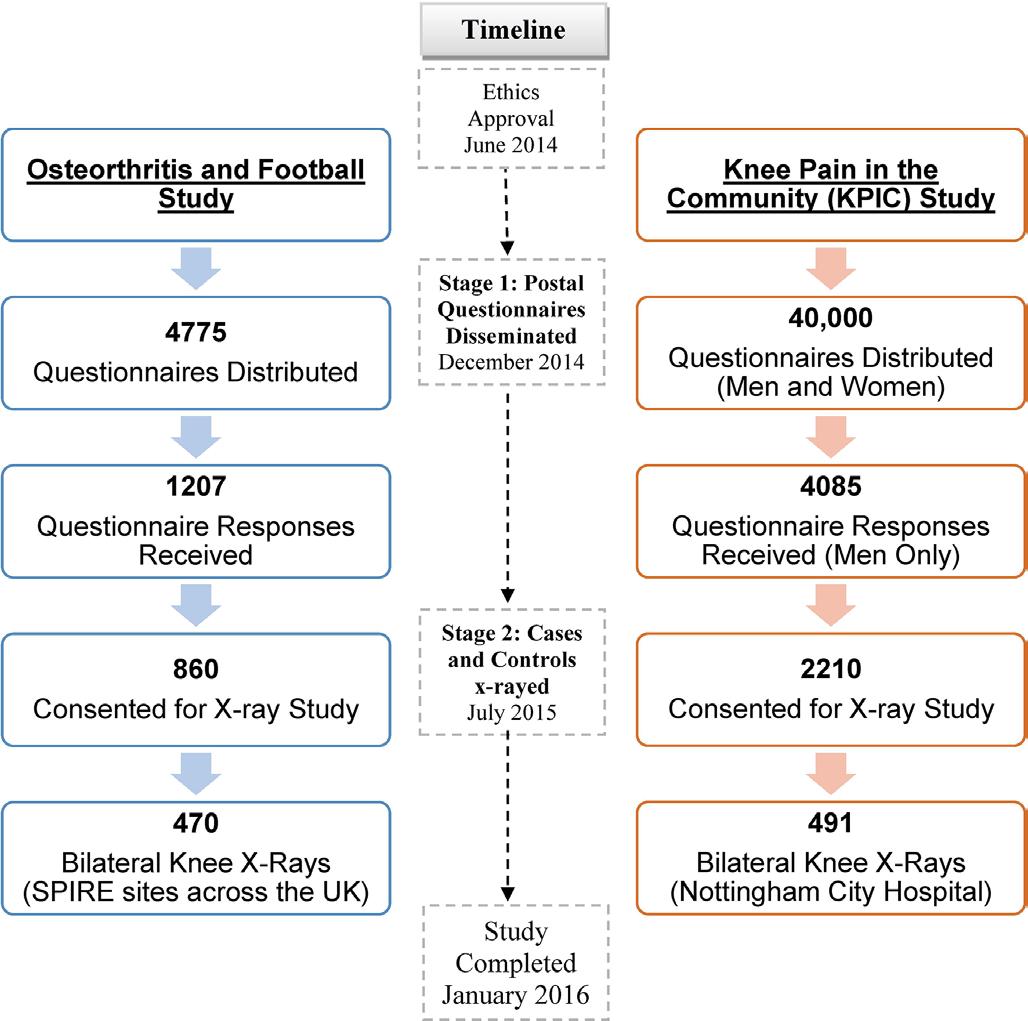
Selection of the ex-footballer and the general population groups.

Of 4775 questionnaires sent to ex-footballers, 1207 responses (25%) were received. Of 40 000 questionnaires sent to men and women in the community (KPIC), 9517 completed questionnaires (24%) were returned, including 4085 men (42.9%) (figure 1).

The mean age of ex-footballers was 59 years (±11.7), 3.9 years younger than the comparison group (62.9±10.4 years), but mean BMI were comparable (table 1). Ex-footballers had a higher prevalence of nodal OA, 2D:4D pattern 3 and body pain (all p<0.01) and were more likely to report constitutionally malaligned knees (varus or valgus) compared with men in the general population. Ex-footballers had fewer comorbidities such as diabetes and cancer (p<0.01) (table 1). Details on missing data for each variable have been provided in online supplementary appendix 1.

10.1136/bjsports-2017-097503.supp1 Supplementary file 1

**Table 1:** Demographics of the footballer and general population groups

	Footballers	General population	p Value
Questionnaires, n	1207	4085	
Age (years), mean (SD)	59.0 (11.7)	62.9 (10.4)	<0.001**
BMI (kg/m^2^), mean (SD)	27.3 (3.2)	27.5 (4.7)	0.139
Right handed, n (%)	1057 (87.6)	3474 (85.0)	0.529
Right lower limb dominance, n (%)	1000 (82.85)	N/A	N/A
Pattern 3 digit ratio, n (%)	733 (60.7)	2237 (54.8)	0.003**
Nodal osteoarthritis, n (%)	86 (7.1)	218 (5.6)	<0.001**
Knee injury, n (%)	778 (64.5)	953 (23.3)	<0.001**
High-risk occupation, n (%)	742 (61.5)	2185 (53.5)	<0.001**
Malalignment, n (%) †			
Constitutional	193 (16.0)	278 (6.8)	<0.001**
Current	289 (24.6)	434 (11.2)	<0.001**
Proportion with change in alignment since 20s, (%)			
Varus	88 (8.9)	132 (3.7)	<0.001**
Valgus	32 (3.2)	42(1.2)	
Body pain, n (%)‡	901 (74.7)	2574 (69.8)	0.001**
Painkillers, n (%) §	423 (35.0)	1289 (31.6)	0.02*
Comorbidities, n (%)¶	355 (29.4)	1868 (45.7)	<0.001**

*p<0.05, **p<0.01.

†Malalignment: right knee varus or valgus deformities (symmetry assumed).

‡Pain reported in any region of the body for most days of the past month.

§Painkillers include non-steroidal anti-inflammatories, opioids, over-the-counter medications and auxiliary medications that have a pain-relieving effect.

¶Comorbidities: diabetes/hypertension/ myocardial infarction/cancer/fibromyalgia.

### Self-reported KOA outcomes

**Figure 2:**
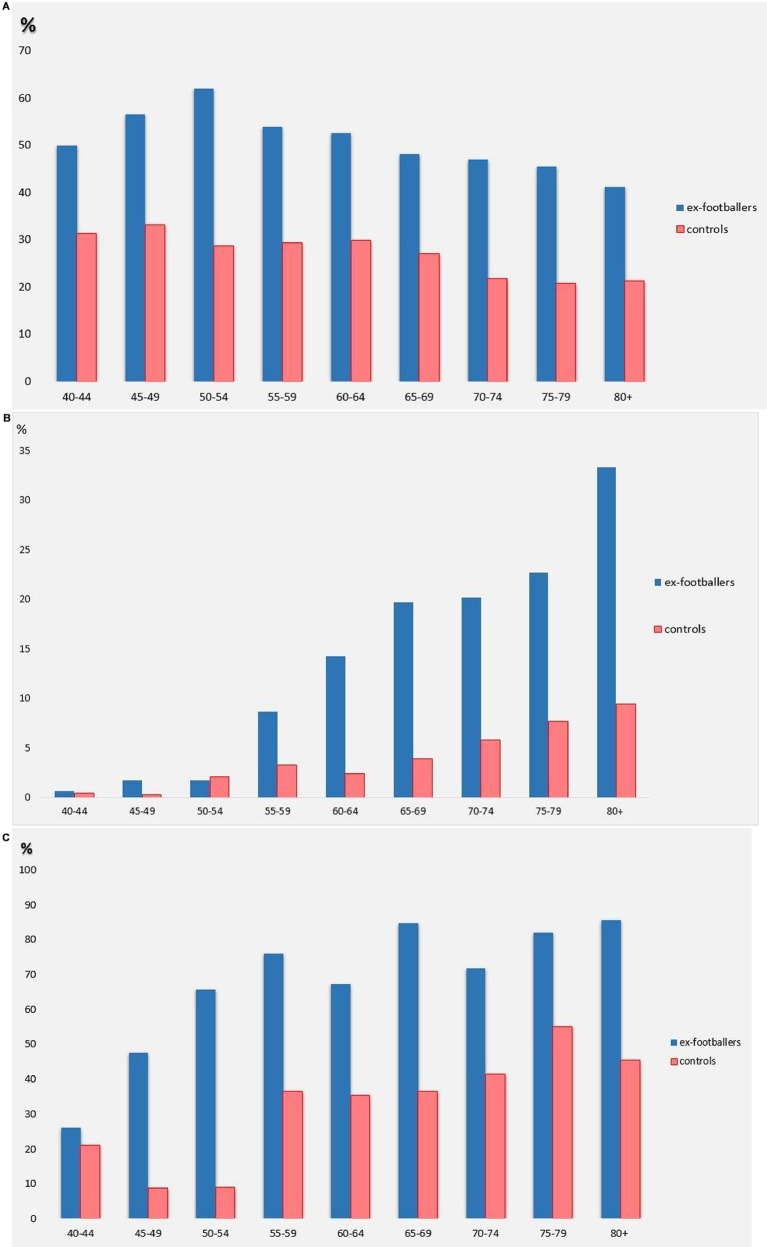
(A) Prevalence of current knee pain by age categories in the ex-footballer and general population; (B) Prevalence of TKR by age categories in the ex-footballer and general population; (C) Prevalence of radiographic knee osteoarthritis by age categories in the ex-footballer and general population.

Overall prevalence of knee pain in ex-footballers was 52.2% compared with 26.9% in the general population (p<0.01). Across all age groups, ex-footballers had more current knee pain than the general population (figure 2A), especially in younger age groups (aged 45–54 years). There was no effect of laterality in knee pain prevalence in the right, left and both knees (see online supplementary appendix 2).

10.1136/bjsports-2017-097503.supp2 Supplementary file 2

After adjusting for age and BMI, the aRR of current knee pain in ex-footballers was 1.91 (95% CI 1.77 to 2.06) compared with the general population. A higher proportion of ex-footballers (28.3%) than men in the general population (12.2%) had received a diagnosis of KOA from a physician (aRR 3.73, 95% CI 3.33 to 4.17). Furthermore, 11.1% of ex-footballers reported TKRs compared with 3.8% of the general population (p<0.01), giving an aRR of 3.61 (95% CI 2.90 to 4.50). The prevalence of TKR by age categories is presented in figure 2B.

### Radiographic findings

Agreement between observers (AS and GSF) examining 21 participants (40 knees) on two occasions using the NLDA was substantial (kappa 0.78), and intra-observer agreement kappas were 1.00 for each observer.

RKOA in any knee using NLDA scoring was present in 64% of footballers and 35.2% of the general population. Ex-footballers had significantly more RKOA in their right, left and both knees (27.2%, 34.6% and 14.4%) compared with the general population (12.2, 10.1% and 5.6%) (see online supplementary appendix 3); 15.7% of ex-footballers had RKOA in their right knee compared with 4.6% in the general population. The prevalence of RKOA by ages is presented in figure 2C.

10.1136/bjsports-2017-097503.supp3 Supplementary file 3

After adjusting for age and BMI, the aRR of RKOA increased from 1.82 to over two times more likely (RR 2.21, 95% CI 1.92 to 2.54) than in the general population. The prevalence of RKOA using KL grading (>3) in any compartment of either knee, was also higher in ex-footballers with an aRR of 2.46 (95% CI 1.89 to 3.22) (table 2).

**Table 2:** Prevalence of knee osteoarthritis and its related outcomes in ex-footballers compared with the general population

	Prevalence, n (%)		RR (95% CI)			
	Footballers	General population	Crude	Model 1	Model 2	Model 3
Self-reported outcomes						
Current knee pain	630 (52.2)	1100 (26.9)	1.94 (1.80 to 2.09)	1.91 (1.77 to 2.06)	1.48 (1.38 to 1.63)	1.50 (1.28 to 1.76)
Physician-diagnosed knee OA	341 (28.3)	500 (12.2)	3.53 (3.15 to 3.96)	3.73 (3.33 to 4.17)	2.69 (2.36 to 3.07)	2.18 (1.73 to 2.77)
Total knee replacement	134 (11.1)	157 (3.8)	2.88 (2.31 to 3.60)	3.61 (2.90 to 4.50)	2.33 (1.84 to 2.95)	2.10 (1.42 to 3.14)
Radiographic outcomes						
Nottingham Line Drawing Atlas (>2 osteophyte and >2 joint space narrowing)	301 (64.0)	173 (35.2)	1.82 (1.58 to 2.08)	2.21 (1.92 to 2.54)	1.91 (1.65 to 2.22)	1.92 (1.66 to 2.23)
Kellgren Lawrence (grade >3)	134 (28.5)	69 (14.1)	2.02 (1.56 to 2.63)	2.46 (1.89 to 3.22)	2.10 (1.58 to 2.80)	2.08 (1.56 to 2.79)
Kellgren Lawrence (grade **>**2)	257 (54.7)	154 (31.4)	2.06 (1.692.53)	2.46 (2.11 to 3.02)	1.99 (1.60 to 2.49)	1.97 (1.58 to 2.46)
Chondrocalcinosis	114 (24.3)	43 (8.8)	2.77 (1.99 to 3.84)	3.41 (2.44 to 4.77)	2.63 (1.84 to 3.75)	2.57 (1.80 to 3.66)

*RR adjusted for football status, age, BMI.

†RR adjusted for football status, age, BMI and injury.

‡RR adjusted for football status, age, BMI, nodal OA, injury, constitutional alignment, high-risk occupation, 2D:4D ratio, nodal OA, comorbidities.

BMI, body mass index; OA, osteoarthritis; RR, relative risk.

Ex-footballers had more RKOA in their left compared with their right knee. They also showed most JSN in the patellofemoral (PF) compartment, whereas the general population had most JSN in the TF compartment (see online supplementary appendix 3). Ex-footballers also had more RKOA in their right and left TF compartment (15.7% and 11.6%) compared with the general population (4.6% and 5%) (see online supplementary appendix 4).

10.1136/bjsports-2017-097503.supp4 Supplementary file 4

Ex-footballers were more likely than the general population to have CC (24.3% vs 8.8%) with an aRR of 3.41 (95% CI 2.44 to 4.77) (table 2), and particularly had more CC in the left knee (see online supplementary appendix 5).

10.1136/bjsports-2017-097503.supp5 Supplementary file 5

Table 3 presents the crude and adjusted risk difference (RD) for each knee OA outcome in the ex-footballers compared with the controls. The absolute measure of association for knee pain suggests that even after adjustment for age and BMI (model 1), 24.46% (21.30%–27.62%) more ex-footballers would present with knee pain compared with the general population. Similarly, even after adjustment for age and BMI, 37.49% (31.82%–43.16%) more ex-footballers would have RKOA using the NLDA compared with the general population.

**Table 3:** Risk differences in knee osteoarthritis and its related outcomes in ex-footballers compared with the general population

Knee OA outcomes	Crude	Model 1	RD **%** (95 % CI) Model 2	Model 3
Current knee pain	25.27 (22.14 to 28.40)	24.46 (21.30 to 27.62)	14.59 (11.32 to 17.87)	14.50 (7.97 to 21.03)
Total knee replacement	7.26 (5.39 to 9.13)	9.53 (7.44 to 11.63)	5.40 (3.63 to 7.17)	4.52 (1.39 to 7.65)
Nottingham Line Drawing Atlas (>2 osteophyte and >2 joint space narrowing)	28.81 (22.75 to 34.86)	37.49 (31.82 to 43.16)	31.11 (24.61 to 37.60)	31.45 (24.87 to 38.03)
Kellgren Lawrence (grade>3)	14.46 (9.35 to 19.57)	18.77 (13.46 to 24.08)	15.33 (9.56 to 21.09)	15.02 (9.17 to 20.88)
Kellgren Lawrence (grade>2)	21.98 (16.26 to 27.71)	27.61 (21.82 to 33.39)	21.21 (14.79 to 27.63)	20.75 (14.22 to 27.28)
Chondrocalcinosis	15.50 (10.89 to 20.11)	18.91 (14.04 to 23.77)	14.69 (9.56 to 19.82)	14.30 (9.18 to 19.41)

*RD adjusted for football status, age, BMI.

†RD adjusted for football status, age, BMI and injury.

‡RD adjusted for football status, age, BMI, nodal OA, injury, constitutional alignment, high-risk occupation, 2D:4D ratio, nodal OA, comorbidities.

BMI, body mass index; OA, osteoarthritis; RD, risk differences.

### Risk factors

Apart from being professional footballers, knee injury, BMI, other high-risk occupation, constitutional knee malalignment and 2D:4D finger ratio were also significantly associated with knee pain, RKOA and TKR (see online supplementary appendix 6).

10.1136/bjsports-2017-097503.supp6 Supplementary file 6

## Discussion

This is the first study comparing the risk of KOA in ex-footballers and the general population. The main findings in footballers are: first, a near twofold increased risk of current knee pain (aRR 1.91, 95% CI 1.77 to 2.06), which was most marked in younger age groups; second, a twofold increased prevalence of RKOA (aRR 2.21, 95% CI 1.92 to 2.54) at all age groups and a threefold increased prevalence of radiographic CC (aRR 3.41, 95% CI 2.44 to 4.77) and third, almost a three times higher requirement for TKR (aRR 3.61, 95% CI 2.90 to 4.50). The major attributable risk factors are knee injury (aRR 1.89 for knee pain, 1.44 for RKOA and 3.32 for TKR), BMI (aRR 1.49 for knee pain and 2.24 for TKR) and nodal OA (aRR 1.46 for knee pain and 1.93 for TKR). However, even after adjusting for injury and other risk factors there is still over a twofold increased risk of KOA outcomes which supports an important role for repetitive microtrauma associated with playing football.

The knee pain prevalence in the general population group accords with previous general population surveys^14 27^ and that in ex-footballers also is similar to previous reports.^2^ Ex-footballers were three times more likely to report a physician-diagnosis of KOA with a prevalence (28.3%) in line with previous questionnaire surveys.^5 6^ In this study, ex-footballers had three times more RKOA and specifically, JSN, in their right TF articulation (15.7%) than the general population (4.6%), which is similar to one study,^9^ but lower than others.^7,8^ This discrepancy may arise from smaller population samples in previous studies, selection bias from recruiting from a single club and radiographic cut-offs using ‘possible’ JSN. When using this cut-off (KL >2), our results remained unchanged with a twofold increased risk of RKOA. Our preferred RKOA definition requiring definite osteophyte and definite narrowing (KL >3) accords with pathological definitions of KOA that require both focal loss of hyaline cartilage and bone hypertrophy.^28^ Furthermore, we used the interval scale NLDA to score narrowing and osteophyte separately, which has several advantages over more commonly used ordinal scale photographic atlases and the composite KL grading system, particularly accounting for wider joint space widths in men and assessing the entire joint (all three compartments) with a global score.^21^ In addition to this, the NLDA was constructed to score changes in the TF and PF compartments of the knee, whereas KL grading has only subsequently been adapted for use in the PF compartment (using the same verbal descriptors as for the combined TF compartments) and was not specifically designed for this purpose originally. Nevertheless, despite these caveats we included the KL grading to permit comparison with other studies that used KL grading to assess radiographic OA severity. The prevalence of CC was significantly higher in ex-footballers (24.3%) compared with the general population (8.8%) and previous population estimates (7%–10%).^29^ Both KOA and prior joint insult are recognised risk factors for CC,^11^ so this increased prevalence is consistent with more biomechanical knee trauma in footballers. Furthermore, concurrence of CC with KOA may associate with greater clinical severity and worse outcomes.^12^ Interestingly, while our general population had more right knee RKOA, ex-footballers had significantly more left knee RKOA, despite the majority indicating right limb dominance. As most football injuries are non-body contact in nature,^3^ this finding might be attributed to playing technique where players cannot respond quickly enough to rapid, unpredictable movements and where rotational strain on the weight-bearing, non-dominant/kicking limb may cause damage.^8^ Additionally, in ex-footballers JSN particularly targeted the PF compartment which is an important component of the knee extensor mechanism that could be stressed by kicking with a partially flexed knee, by constitutional malalignment (which was increased in footballers (table 1)) or by torsional movements that influence patellar tracking, all of which may initiate PF degeneration.^30^

Ex-footballers reported more knee pain at all ages, and this was particularly marked in younger age groups (figure 2A). Having adjusted for all significant risk factors, including injury, ex-footballers still showed increased risks of knee pain, RKOA and TKR suggesting lasting damage from repetitive microtrauma sustained over the course of their footballing career. This is the first nationwide study to examine structural and person-centred outcomes relating to KOA in ex-footballers. The reported degree of increased adjusted risk (at least doubled) is in the order required by many national bodies to recognise KOA as an industrial disease for professional football. Importantly, the study also identifies risk factors for KOA in footballers and in particularly, key modifiable risk factors, such as knee injury, being overweight/obese and undertaking high-risk occupations after retiring from football (see online supplementary appendix 6).

## Caveats

There are several limitations to this study. First, the use of self-reported questionnaires might involve recall bias and possible misclassification of self-reported outcomes.^31^ A structured clinical enquiry and assessment of every participant would have been preferable but was impractical due to logistics (UK nationwide survey) and limited resources. Nevertheless, we used validated instruments designed for questionnaire use and involved ex-footballers, patients and general public volunteers to help optimise clarity and ease of use of the questionnaire. Second, the suboptimal response rate to the questionnaire and subsequently the ex-footballers and general population subsamples indicating willingness to have knee X-rays, could have resulted in selection bias. Unfortunately, we could not compare simple demographics or health records of responders and non-responders in either group due to data protection of externally held databases and patient confidentiality in the general population group. However, although knee pain positive individuals might be more likely to respond, this bias should be present in both groups. Furthermore, this is a large cross-sectional study and given that the prevalence of knee pain, RKOA, CC and risk factors in the general population generally aligned with results from other population-based studies, it seems unlikely that the estimates are unduly biased. Third, although we surveyed ex-footballers over a wide area of the UK, for logistical reasons the general population was recruited from just one region. Nevertheless, the East Midlands was a major coal-mining region, a male occupation with an established risk of RKOA.^4^ Thus, any regional bias in the general population would have been expected to inflate rather than lower the prevalence of KOA, making the demonstrated increased risk in ex-footballers even more confident. Fourth, although radiographs are widely used to assess structural KOA in population-based studies, they are insensitive to soft tissue and other changes indicative of early KOA that can be identified by alternative imaging techniques such as MRI.^32^ Therefore, we are likely to have underestimated the true prevalence of structural OA in both ex-footballers and the general population. However, use of MRIs in this study was not financially or logistically viable. Another caveat is that while the authors were able to include known risk factors of KOA such as age, BMI and injury, there are still unmeasured confounders such as physical activity or unknown confounders that might contribute to the increased KOA outcomes in ex-footballers compared with the general population.

In summary, the prevalence of knee pain and RKOA in ex-footballers is twice as high as in men in the general population and ex-professional players develop knee pain earlier and require three times more knee joint replacements. This study took into account other risk factors for knee OA and demonstrates that professional football is a significant risk factor for the development of the symptoms (knee pain), structural change (RKOA, CC) and requirement for TKR. Knee injury is a major attributable risk factor for each of these outcomes. These findings have important consequences for football associations/unions and stakeholders for whom the health of retired and current professional footballers is paramount.

**What are the findings?**

- This is the first large-scale cross-sectional study to compare outcomes of knee pain, radiographic osteoarthritis and requirement for knee arthroplasty in ex-footballers to men in the general population. The prevalence of all knee outcomes was almost two to three times higher in ex-footballers even after adjustment for known risk factors including significant knee injury.
- The prevalence of knee pain by age reached its peak 10–15 years earlier in ex-footballers compared with men in the general population.
- Knee injury, a high body mass index and a high-risk occupation (postretirement from football) are the main attributable risk factors for the increases in knee pain, radiographic osteoarthritis and knee replacement in ex-professional footballers.

**How might it impact on clinical practice in the future?**

The results indicate modifiable risk factors such as obesity and significant knee injuries which can be better managed through self-education, early treatment, appropriate rehabilitation before return to play, etc in order to reduce knee osteoarthritis outcomes. This is an important finding for football associations/unions and stakeholders for whom the health of retired and current professional footballers is a priority.
