# Supplementary material for: Prevalence of knee pain, radiographic osteoarthritis and arthroplasty in retired professional footballers compared with men in the general population: a cross-sectional study
Source: Br J Sports Med. 2017 Nov 3;52(10):678–83. doi: 10.1136/bjsports-2017-097503 (PMC5931242; doi:10.1136/bjsports-2017-097503)
Supplement: Supplementary file 1 [file bjsports-2017-097503supp001.docx]

Appendix 1: Missing data for variables in Table 1.

|  | Missing Data, n (%) | |
| --- | --- | --- |
| Questionnaire *n* | Footballers Total n=1207 | General Population  Total n= 4085 |
| Age | 5 (0.4) | 10 (0.2) |
| BMI | 25 (2.1) | 137 (3.4) |
| Right Handed, n (%) | 19 (1.6) | 151 (3.7) |
| Right limb dominance, n (%) | 0 | N/A |
| Pattern 3 Digit Ratio, n (%) | 55 (4.6) | 278 (6.8) |
| Nodal OA, n (%) | 0 | 0 |
| Knee Injury, n (%) | 0 | 0 |
| High Risk Occupation, n (%) | 0 | 0 |
| Malalignment, n (%) ^†^ |  |  |
| Constitutional | 28 (2.3) | 237 (5.8) |
| Current | 31 (2.6) | 202 (4.9) |
| Body Pain, n (%) ^‡^ | 0 | 0 |
| Painkillers, n (%) ^#^ | 0 | 0 |
| Comorbidities, n (%)^^^ | 0 | 0 |
