# Supplementary material for: Prevalence of knee pain, radiographic osteoarthritis and arthroplasty in retired professional footballers compared with men in the general population: a cross-sectional study
Source: Br J Sports Med. 2017 Nov 3;52(10):678–83. doi: 10.1136/bjsports-2017-097503 (PMC5931242; doi:10.1136/bjsports-2017-097503)
Supplement: Supplementary file 2 [file bjsports-2017-097503supp002.docx]

**Appendix 2: Proportional Bar Graph showing knee pain distribution in right, left and both knees in the ex-footballers and the general population.**

LK

BK

RK

LK

BK

RK

*# RK: right knee; LK: left knee; BK: bilateral (both knees)*
