# Supplementary material for: Prevalence of knee pain, radiographic osteoarthritis and arthroplasty in retired professional footballers compared with men in the general population: a cross-sectional study
Source: Br J Sports Med. 2017 Nov 3;52(10):678–83. doi: 10.1136/bjsports-2017-097503 (PMC5931242; doi:10.1136/bjsports-2017-097503)
Supplement: Supplementary file 3 [file bjsports-2017-097503supp003.docx]

**Appendix 3. Definite RKOA (NLDA) in ex-footballers versus the general population (i) and compartmental JSN in the right knee (ii) and left knee respectively (iii)**

BK

LK

RK

BK

LK

RK

*# RK: right knee; LK: left knee; BK: bilateral (both knees)*

3

1

2

3

1

2

*# 1: Medial Tibiofemoral; 2: Lateral Tibiofemoral; 3: Patellofemoral*

2

3

2

1

3

1

*# 1: Medial Tibiofemoral; 2: Lateral Tibiofemoral; 3: Patellofemoral*
