# Supplementary material for: Prevalence of knee pain, radiographic osteoarthritis and arthroplasty in retired professional footballers compared with men in the general population: a cross-sectional study
Source: Br J Sports Med. 2017 Nov 3;52(10):678–83. doi: 10.1136/bjsports-2017-097503 (PMC5931242; doi:10.1136/bjsports-2017-097503)
Supplement: Supplementary file 4 [file bjsports-2017-097503supp004.docx]

**Appendix 4: Distribution of RKOA (KL) in Ex-Footballers compared to the general population.**
