# Supplementary material for: Prevalence of knee pain, radiographic osteoarthritis and arthroplasty in retired professional footballers compared with men in the general population: a cross-sectional study
Source: Br J Sports Med. 2017 Nov 3;52(10):678–83. doi: 10.1136/bjsports-2017-097503 (PMC5931242; doi:10.1136/bjsports-2017-097503)
Supplement: Supplementary file 5 [file bjsports-2017-097503supp005.docx]

**Appendix 5: Distribution of Chondrocalcinosis in Ex-Footballers compared to the general population.**


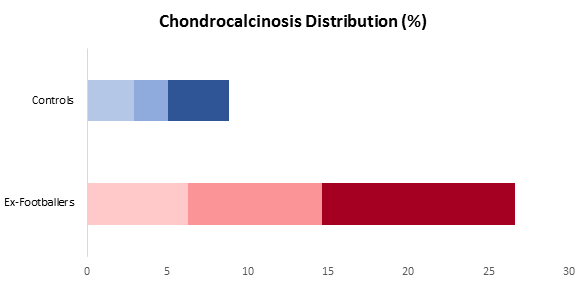


Ex-Footballers

General Population

BK

BK

LK

LK

RK

RK

*# RK: right knee; LK: left knee; BK: bilateral (both knees)*
