# Supplementary material for: Prevalence of knee pain, radiographic osteoarthritis and arthroplasty in retired professional footballers compared with men in the general population: a cross-sectional study
Source: Br J Sports Med. 2017 Nov 3;52(10):678–83. doi: 10.1136/bjsports-2017-097503 (PMC5931242; doi:10.1136/bjsports-2017-097503)
Supplement: Supplementary file 6 [file bjsports-2017-097503supp006.docx]

**Appendix 6. Risk factors associated with knee pain (KP), radiographic knee OA (RKOA) and total knee replacement (TKR)**

| **Risk Factor** | aRR Current Knee Pain (95% CI) | aRR RKOA with NLDA ǂ (95% CI) | aRR TKR  (95% CI) |
| --- | --- | --- | --- |
| **Age** | 0.99  (0.98 – 0.99) | 1.03  (1.02-1.04)* | 1.07  (1.10-1.09)* |
| **BMI** (overweight)** | 1.49  (1.34 – 1.65)* | 1.13  (0.97-1.31) | 2.24  (1.47 – 3.42)* |
| **High Risk Occupation** | 1.29  (1.19 – 1.40)* | 1.08  (0.96-1.23) | 1.37  (1.01 – 1.84)* |
| **Alignment in 20’s** | 1.15  (1.03-1.28)* | 1.17  (0.98-1.38) | 3.35  (2.41-4.68)* |
| **Knee Injury** | 1.89  (1.74-2.06)* | 1.44  (1.25-1.66)* | 3.32  (2.45 – 4.51)* |
| **Digit Ratio** | 1.00  (0.93-1.08) | 0.89  (0.78-1.02) | 0.93  (0.7 – 1.25) |
| **Nodal OA** | 1.46  (1.29-1.65)* | 1.03  (0.84-1.27) | 1.93  (1.33 -2.80)* |

aRR: adjusted relative risk (RR), adjusted for footballer status and other risk factors in the table; CI: confidence interval

*Denotes statistical significance (p<0.05)

**BMI categories were created using WHO guideline cut offs for those overweight BMI >25

ǂ NLDA cut off was defined as definite osteophyte (global osteophyte score > 2) and definite narrowing (JSN > 2).
